# Human Menstrual Blood-Derived Mesenchymal Stem Cells as Potential Cell Carriers for Oncolytic Adenovirus

**DOI:** 10.1155/2017/3615729

**Published:** 2017-07-11

**Authors:** R. Moreno, L. A. Rojas, Felip Vilardell Villellas, Vanessa Cervera Soriano, J. García-Castro, C. A. Fajardo, R. Alemany

**Affiliations:** ^1^Virotherapy and Gene Therapy Group, ProCure Program, Translational Research Laboratory, Instituto Catalan de Oncología-IDIBELL, Barcelona, Spain; ^2^Servei d'Anatomia Patològica, Hospital Universitari Arnau de Vilanova i Institut de Recerca Biomèdica de Lleida, Lleida, Spain; ^3^Genomics and Cytomics Unit, Bellvitge Biomedical Research Institute (IDIBELL), Barcelona, Spain; ^4^Cellular Biotechnology Laboratory, Institute of Health Carlos III (ISCIII), Majadahonda, Madrid, Spain

## Abstract

Antitumor efficacy of systemically administered oncolytic adenoviruses (OAdv) is limited due to diverse factors such as liver sequestration, neutralizing interactions in blood, elimination by the immune system, and physical barriers in tumors. It is therefore of clinical relevance to improve OAdv bioavailability and tumor delivery. Among the variety of tumor-targeting strategies, the use of stem cells and specifically bone marrow-derived mesenchymal stem cells (BM-MSCs) is of particular interest due to their tumor tropism and immunomodulatory properties. Nonetheless, the invasive methods to obtain these cells, the low number of MSCs present in the bone marrow, and their restricted in vitro expansion represent major obstacles for their use in cancer treatments, pointing out the necessity to identify an alternative source of MSCs. Here, we have evaluated the use of menstrual blood-derived mesenchymal stem cells (MenSCs) as cell carriers for regional delivery of an OAdv in the tumor. Our results indicate that MenSCs can be isolated without invasive methods, they have an increased proliferation rate compared to BM-MSCs, and they can be efficiently infected with different serotype 5-based capsid-modified adenoviruses, leading to viral replication and release. In addition, our in vivo studies confirmed the tumor-homing properties of MenSCs after regional administration.

## 1. Introduction

Oncolytic adenoviruses (OAdv) have been extensively studied and tested in clinical trials involving a variety of cancer types. Results from those clinical trials revealed good toxicological and safety profile, but modest efficacy [1]. OAdv face numerous challenges that hinder their successful application. Upon systemic administration, OAdv can be filtered and retained in normal tissues, especially the liver. The immune system can recognize the adenovirus in the bloodstream leading to its elimination. Moreover, to exit the bloodstream and enter the extracellular space, virus particles have to overcome the abnormal tumor vascular system [2] and the elevated interstitial fluid pressure [3]. Finally, the tumor microenvironment contains several barriers that limit drug penetration and delivery, such as an extracellular matrix (ECM) rich in proteins, hyaluronic acid, proteoglycans, and stromal cells [4, 5].

The use of cell carriers to deliver oncolytic viruses to primary tumors and metastases addresses many of these obstacles. In this regard, some types of stem cells have garnered significant interest due to their capability to migrate specifically toward tumors [6, 7]. Thus, systemic administration of autologous and allogeneic stem cells loaded with the oncolytic virus could evade the filtering organs and the immune system and cross the endothelial barrier.

Mesenchymal stem cells (MSCs) are adult stem cells, which can be isolated and expanded ex vivo from a great variety of sources and species [8]. MSCs are considered to have low immunogenicity owing to their unique immunologic characteristics: MSCs express low levels of HLA class I, but neither HLA class II nor CD40, CD80, and CD86 costimulatory molecules on their surface [9]. Moreover, MSCs induce little proliferation of allogeneic lymphocytes and modulate the activity of cytotoxic T cells, dendritic cells, and B cells [10, 11]. In addition, MSCs are known to migrate to sites of injury and inflammation, which are two characteristics of the tumor microenvironment [12, 13]. All these attributes make MSCs particularly appealing as cell carriers for oncolytic viruses. In fact, proof of principle of MSCs as cell carriers for OAdv has been demonstrated in several animal models [14–16], and their efficacy has been evaluated in a clinical trial for cancer treatment [17, 18].

The bone marrow represents the main and most frequent source for MSC isolation and amplification. Nonetheless, the invasive methods used to obtain these cells, the low number of MSCs present in bone marrow (0.001–0.01% total nuclear cells [19]), and their slow and restricted in vitro expansion represent major obstacles for their use in cancer treatment. It would be therefore preferable to identify an alternative source of MSCs that allow an easy isolation without clinical intervention or hospitalization and with a high content of cells to minimize in vitro expansion.

In 2004, Chan and coworkers showed the existence of a mesenchymal cell population in the human endometrium which represents approximately 1% of endometrial cells [20]. Later on, Patel et al. demonstrated that the shed menstrual blood and tissue represents a rich source for these endometrium mesenchymal stem cells, suggesting that it was not necessarily an invasive procedure (hysterectomia or biopsia) for their obtention [21]. It was further confirmed that menstrual blood-derived mesenchymal stem cells were positive for mesenchymal stem cell markers, can be rapidly expanded without chromosomal aberration, and can differentiate into a variety of cell types from the three germ layers (feature characteristic of mesenchymal stem cells) [22]. In 2013, the group of Filippo Rossignoli compared MSCs isolated from different origins (bone marrow, peripheral blood, dental pulp, umbilical cord, adipose tissue, and decidual tissue) and determined that MenSCs resemble more closely to bone marrow-derived MSCs than MSCs obtained from other tissues. Furthermore, they demonstrated that MenSCs have the shortest population doubling time, the highest clonogenic efficacy, and the largest number of in vitro passages before becoming senescent [23]. More recently, MenSCs have been extensively characterized in vitro and in vivo and their potential for cellular therapy has been described [24–26]. Altogether, these features point out that menstrual blood represents an efficient and ethically accepted source of MSCs for clinical treatments.

Currently, the use of MenSCs has been applied to the treatment of multiple sclerosis, Duchenne muscular dystrophy, and more recently, heart failure and liver cirrhosis (reviewed in [26]). Here, we propose the use of MenSCs as cell carriers for OAdv and cancer treatment.

In this study, we show the proliferative potential of menstrual blood-derived mesenchymal stem cells and demonstrate that MenSCs can be efficiently infected with different Ad5-based capsid-modified adenoviruses, leading to viral replication and release of new viral progeny. Finally, using in vivo imaging studies in mice, we demonstrate that MenSCs loaded with an oncolytic adenovirus (OAdv) present tumor tropism upon regional administration.

Our results indicate that MenSCs represent a promising candidate as cell carriers for oncolytic adenovirus delivery to human tumors.

## 2. Materials and Methods

### 2.1. Cell Culture

#### 2.1.1. Isolation of Human Menstrual Blood-Derived Mesenchymal Stem Cells

Menstrual blood (1–5 ml) was collected from healthy female donors (*n* = 7), aged 23–42 years, on the first 3 days of the menstrual phase using a menstrual cup (Mooncup, Brighton, UK). The protocol and cell donation for research purposes were approved by the Institutional Ethics Committee, and written consent was signed for each donor. Blood samples were transferred to a sterile 50 ml centrifuge tube with conical bottom, filled with sterile phosphate buffered saline (PBS, Life Technologies, Carlsbad, CA, USA), and centrifuged at 1500 rpm for 5 minutes. The pellet was resuspended in *α*-MEM (Life Technologies) containing 20% fetal bovine serum, 1% penicillin/streptomycin, and 1% gentamicin/amphotericin B (all from Life Technologies) and seeded overnight in culture flasks (Corning Inc., Corning, NY) at 37°C in a 5% CO_2_ atmosphere.

The next day, cell monolayer was washed once with PBS to remove nonadherent cells and cellular debris and replaced with fresh medium. Finally, MenSCs were amplified for 7–24 days (passage 0), substituting the culture medium every 3-4 days, until 75%–90% confluent, and then passaged periodically by detachment (0.25% trypsin-1 mM EDTA in PBS (Life Technologies)) after achieving a subconfluent monolayer.

#### 2.1.2. Bone Marrow-Derived Mesenchymal Stem Cells

BM-MSCs were obtained from the American Type Culture Collection (ATCC, Manassas, VA) and maintained with *α*-MEM (Life Technologies) supplemented with 15% fetal bovine serum and 1% penicillin/streptomycin (all from Invitrogen) at 37°C, 5% CO_2_.

#### 2.1.3. Cell Lines

A549 (human lung adenocarcinoma), SKmel28 (human melanoma), and Panc-1 (human pancreatic adenocarcinoma) were obtained from the American Type Culture Collection (ATCC, Manassas, VA). NP-18 pancreatic tumor cell lines were established in our laboratory [27]. All tumor cell lines were maintained with Dulbecco's modified Eagle's medium supplemented with 5 or 10% fetal bovine serum and 1% penicillin/streptomycin (all from Invitrogen) at 37°C, 5% CO_2_. All cell lines were routinely tested for mycoplasma presence.

### 2.2. Phenotypic Characterization

MenSCs (passage 3) were analyzed by flow cytometry for expression of the different cell surface markers. Fluorescein isothiocyanate- (FITC-) conjugated antibodies against CD29 (Immunostep, Salamanca, Spain), CD90 (eBioscience, San Diego, CA, USA), HLA-DR, CD14, CD34, CD83, CD86 (BD Biosciences, San Jose, CA, USA), CD40 (Biolegend, San Diego, CA, USA), phycoerythrin- (PE-) conjugated antibody against CD73 (BD biosciences), CD105a (eBiosciences) CD133 (Miltenyi Biotec, Bergisch Gladbach, Germany), and Alexa Fluor 700-conjugated antibody against CD44 (BD Biosciences) were used. The corresponding fluorescent, isotype-matched negative control antibodies defined background staining.

Harvested MenSCs were washed with PBS containing 1% bovine serum albumin (BSA, Sigma-Aldrich, St. Louis, MO) and 0.1% sodium azide (Sigma-Aldrich). Aliquots of 2 × 10^5^ cells were next incubated on ice with the corresponding antibody, at the concentration recommended by the manufacturer, for 20 min and under light protection. A Gallios cytometer (Beckman Coulter, Brea, CA, USA) was used, and 10,000 events were analyzed for each sample. FlowJo v7.6.5 (Tree Star Inc., San Carlos, CA, USA) software was used for the analysis of the data.

### 2.3. In Vitro Differentiation of MenSCs

MenSCs (passage 3) were plated in 12-well plates with *α*-MEM containing 20% fetal bovine serum, 1% penicillin/streptomycin, and 1% gentamicin/amphotericin B and used for the differentiation study when reached at 80%–90% of confluence.

Adipogenic, chondrogenic, and osteogenic differentiation were induced by culturing the cells with commercial differentiation media (all from Life Technologies). To confirm correct differentiation after 14–21 days in culture, oil red O staining for lipid droplets, alcian blue staining for sulfated proteoglycan-rich matrix, and alizarin red staining to detect calcified extracellular matrix deposits were performed to confirm correct adipogenic, chondrogenic, and osteogenic differentiation, respectively. Nondifferentiated but stained MenSCs as stained controls were also included.

### 2.4. Colony-Forming Unit (CFU) Assay

MenSCs from 5 different donors (passages 4–6) and BM-MSCs (passage 5) were seeded at clonal density of 500–1000–2000 cells/cm^2^ in 6-well cell culture plates. After 10 days in culture, cells were washed with PBS, fixed with methanol for 10 minutes, and stained with 0.5% crystal violet for 20 minutes at room temperature and the corresponding colonies were counted.

### 2.5. Population Doubling Time

The population doubling time (DT) was assessed by seeding 10,000 MenSCs (from 5 different donors, passages 4–6) or BM-MSCs (passage 5) in 6-well cell culture plates with complete medium and counting the cell number at days 3, 5, 7, and 10 of culture. The DT was determined using a doubling time calculation software equation (Roth V. 2006 http://www.doubling-time.com/compute.php).

### 2.6. Generation of Recombinant Adenoviruses and Adenoviral Vectors

Adenoviral vectors AdGL (wild-type capsid), AdGLRGD (insertion of Arg-Gly-Asp (RGD) in the HI-loop of the fiber knob), and AdGLK (replacement of the KKTK fiber shaft heparan sulfate glycosaminoglycan-binding domain with an RGDK motif) expressing the EGFP-luciferase fusion protein cassette [28, 29] and the oncolytic adenovirus ICOVIR15 [30] have been previously described.

### 2.7. Viral Infectivity Assays

MenSCs (passage 3) were seeded in 24-well plates (5 × 10^4^ cells/well) and infected with the adenoviral vectors AdGL, AdGL-RGD, and AdGLK at different multiplicity of infection (MOI 25, 10, 5, and 1 TU/cell) in triplicates during 24 hours at 37°C. After this time, cells were trypsinized and resuspended in FACS buffer (PBS 10% FBS, 0.01% sodium azide), and 5000 events were analyzed by flow cytometry.

### 2.8. Flow Cytometry Analysis of Coxsackievirus-Adenovirus Receptor (CAR) and Integrin Expression

MenSCs (1 × 10^5^ cells/sample) (passage 3) were harvested by trypsin digestion and labeled with either mouse monoclonal anti-CAR RmcB (Upstate Biotechnology, Lake Placid, NY, USA) or mouse monoclonal anti-*α*v-integrin L230 (Enzo Life Sciences, Farmingdale, NY, USA) for 1 h at 4°C. Subsequently, cells were incubated with Alexa Fluor 488-labeled goat anti-mouse IgG (Life Technologies) for 1 h at 4°C. A background control incubated only with secondary antibody was also included. Finally, samples were analyzed by flow cytometer.

### 2.9. Viral Production Assay

MenSCs, BM-MSCs (1 × 10^5^ cells per well in 24-well plates, both at passage 4) and A549 cells (2 × 10^5^ cells per well in 24-well plates) were infected at a MOI of 50 TU/cell (MSCs) and 10 TU/cell (A549). Four hours after infection, medium was removed and cells were washed thrice with PBS and incubated with fresh medium. At the indicated time points of postinfection (0, 24, 48, and 72 h), a small fraction of the supernatant (SN) was collected, and the cells and the medium were harvested and frozen-thawed three times to obtain the cell extract (CE). Viral titers were determined in triplicate by an antihexon staining-based method in HEK293 cells [31].

### 2.10. In Vitro Cytotoxicity Assays

MenSCs (passage 4) were infected with ICOVIR15 at a MOI of 50 TU/cell for 24 h. The next day, infected MenSCs were washed thrice with PBS and cocultured with tumor cell lines (A549, Panc-1, NP-18, and SKmel28) at different MenSC : tumor cell ratios ranging from 1 : 1 to 0.001 : 1. At day 5 of coculture, plates were washed with PBS and stained for total protein content (bicinchoninic acid assay; Pierce Biotechnology, Rockford, IL) and absorbance was quantified. The ratio MenSC : tumor cell required to produce 50% tumor cell growth inhibition (IC_50_) was determined from dose-response curves by standard nonlinear regression (GraphPad Prism; GraphPad Inc., La Jolla, CA).

### 2.11. MSC Staining with DiR

To allow in vivo tracking of MSCs, BM-MSCs and MenSCs (both at passage 7) were stained with XenoLight DiR, a near-IR lipophilic membrane dye (PerkinElmer, Waltham, MA, USA). MSCs were incubated with 320 *μ*g/ml of DiR for 30 min at 37°C according to the manufacturer's protocol.

The DiR-labeled MSCs were spinned down for 5 min at 1000 rpm, and cell pellets were resuspended in PBS. This procedure was repeated twice to ensure complete removal of any unbound dye.

### 2.12. In Vivo Tumor-Homing Studies

In vivo studies were performed at the ICO-IDIBELL facility (Barcelona, Spain) AAALAC unit 1155 and approved by IDIBELL's Ethical Committee for Animal Experimentation.

Subcutaneous xenograft tumors were established by injection of 5 × 10^6^ A549 cells into the flanks of 9-week-old female athymic *nu/nu* mice. When tumors reached 400 mm^3^, 1 × 10^6^ DiR-labeled MenSCs (passage 7) were administered intraperitoneally or intratumorally in mice (*n* = 3). Fluorescence images (using 10 nm excitation and 780 nm emission filters) were monitored at 1 h, 24 h, 48 h, and 5 days postadministration which allowed MSC-homing determination using IVIS Lumina bioimaging system (PerkinElmer). Images were analyzed with IVIS Living Image (PerkinElmer) software. Regions were manually drawn around the tumors.

The same model was used to evaluate the effect of OAdv infection in MSC tumor tropism. When tumors reached 400 mm^3^, mice (experimental day 0) were randomized (*n* = 5 animals per group) into the following groups: PBS, BM-MSCs (previously labeled with DiR); MenSCs (previously labeled with DiR); BM-MSCs-OAdv (BM-MSCs previously infected with ICOVIR15 at MOI 50 for 2 h and labeled with DiR); and MenSCs-OAdv (MenSCs previously infected with ICOVIR15 at MOI 50 for 2 h and labeled with DiR). Animals received a single intraperitoneal administration of PBS, 1 × 10^6^ BM-MSCs; BM-MSCs-OAdv; MenSCs or MenSCs-OAdv. Fluorescence images monitored at 24 h, 48 h, and 72 h postadministration. Images were analyzed with IVIS Living Image (PerkinElmer) software. Regions were manually drawn around the tumors.

### 2.13. Statistical Analysis

Statistical comparisons between groups were performed using the Mann–Whitney *U* test (2-tailed) when *n* > 3. Statistical significance was established as *P* < 0.05. Data are presented as the mean ± SD. All statistical analyses were calculated with the GraphPad Prism software.

## 3. Results

### 3.1. Isolation and Characterization of Human MenSCs

Menstrual blood (1–5 ml) was collected from healthy female donors (*n* = 7) aged 23–42 years on the first 3 days of the menstrual phase, and mesenchymal stem cells were isolated. After one week in culture, adherent cells exhibited a spindle-shaped fibroblast-like morphology reaching confluence after 7–24 days of culture (Supplementary Fig. 1A available online at https://doi.org/10.1155/2017/3615729).

Using commercially available media, MenSCs were induced to differentiate into 3 mesodermal lineages (adipogenic, chondrogenic, and osteogenic) in order to examine their multipotentiality. After 21 days in culture with the different media, lipid vesicles were observed by phase-contrast light microscopy and contrasted with oil red O stain (Supplementary Fig. 1B, left). Alcian blue staining confirmed the presence of sulfated proteoglycan-rich matrix on the chondrogenic MenSC differentiation (Supplementary Fig. 1C, left). Finally, differentiation of MenSCs into osteoblasts was confirmed after cell morphology changed from a spindle-shaped to a cuboidal-like phenotype, forming cell aggregates, and calcium deposition was observed by staining these cells with alizarin red (Supplementary Fig. 1D, left).

MenSCs were also characterized by the expression of several mesenchymal stem cell antigens (CD29, CD90, CD44, CD73, and CD105a) and the absence of hematopoietic markers (CD34, HLA-DR, CD133, and CD14) and costimulatory proteins (CD40, CD83, and CD86) (Supplementary Fig. 2).

Altogether, these results are in agreement with the criteria to define human MSCs established by the International Society for Cellular Therapy (ISCT) [32].

### 3.2. Growth Characteristics of MenSCs

**Figure 1:**
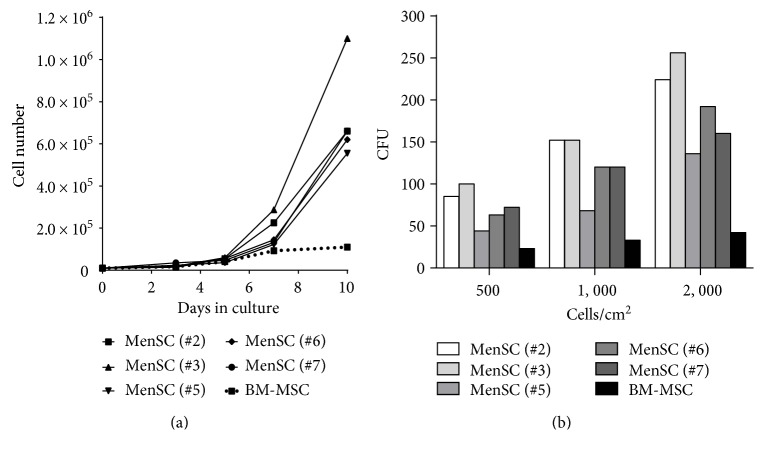
Growth potential of MenSCs. (a) 10,000 MenSCs (from 5 different donors) or BM-MSCs were seeded in 6-well plates with complete medium, and the cell number was counted at days 3, 5, 7, and 10 of culture. (b) Colony-forming unit (CFU) efficiencies of MenSCs (from 5 different donors) and BM-MSCs, plated at a 500, 1000, and 2000 cells/cm^2^ in 6-well plates. After 10 days of culture, cells were stained with crystal violet and the corresponding colonies were counted.

To compare differences in growth potential between MenSCs and BM-MSCs, we first determined the population doubling time (DT) of MenSCs from five donors and commercial BM-MSCs. All MenSCs presented a significantly reduced population DT (39.7 ± 2.3 h), compared with BM-MSCs (63.6 h) (Figure 1(a)), without correlation between the donor age and the DT (linear regression donor age versus DT *r*^2^ = 0.6).

Furthermore, a colony-forming unit (CFU) assay was performed by seeding the MenSC lines and BM-MSCs at different cell densities. The clonogenic ability of all MenSC cell lines was significantly higher than that of BM-MSCs independently of the initial cell density (ratio MenSC/BM-MSC colonies of 3.2 ± 0.9, 3.7 ± 1.0, and 4.6 ± 1.1 when seeding at 500, 1000, and 2000 cells/cm^2^, resp.) (Figure 1(b)).

These data demonstrate that MenSCs have a greater growth potential than BM-MSCs, not only because of their higher clonogenic capability but also because of their shorter population DT and faster cell growth kinetics.

### 3.3. Adenovirus Infection and Replication in MenSCs

**Figure 2:**
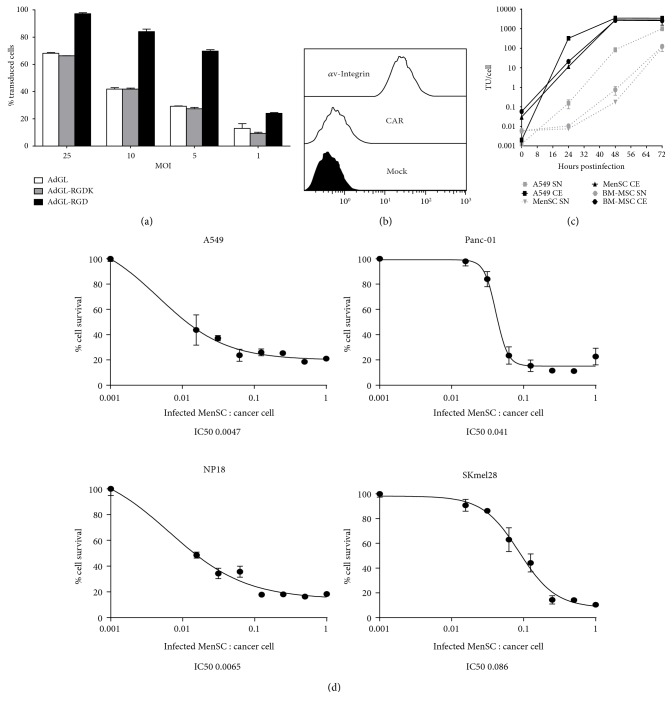
MenSC infection and viral replication. (a) Infectivity assay in MenSCs. MenSCs (5 × 10^4^ cells/well) were infected for 24 hours at 37°C with AdGL, AdGLRGD, and AdGLK at different multiplicities of infection (25, 10, 5, and 1 TU/cell). Infected cells were analyzed by flow cytometry. Results are shown as the % of infected cells (green cells). Mean values (*n* = 3) ± SD are represented. (b) MenSCs were analyzed by flow cytometry for the expression of the main adenovirus type 5 receptors. MenSCs express *α*v-integrins but do not express the coxsackievirus B and adenovirus receptor (CAR). (c) OAdv production kinetics and release in A549 cells, BM-MSCs, and MenSCs. Extracellular (SN) and total virus produced (CE) were measured at the indicated time points. Mean values (*n* = 3) ± SD are plotted. (d) Cytotoxicity analysis of OAdv produced by MenSCs. A549, Panc-01, NP-18, and SKmel28 cancer cell lines were cocultured with previously infected MenSCs (MOI of 50 TU/cell of ICOVIR15 for 24 h before coculture) at different infected MenSCs : cancer cell ratios. At day 5 of coculture, cell survival was measured and IC50 values calculated. Mean values (*n* = 3) ± SD are plotted.

To determine the permissiveness of MenSCs to adenoviral infection and replication, several experiments were performed. We first evaluated the efficacy of three different capsid-modified adenovirus vectors to infect the mesenchymal cells. MenSCs were infected with different amounts of AdGL (wild-type capsid), AdGLRGD (insertion of Arg-Gly-Asp (RGD) in the HI-loop of the fiber knob), and AdGLK (replacement of the KKTK fiber shaft heparan sulfate glycosaminoglycan-binding domain with an RGDK motif). As shown in Figure 2(a), while wild-type capsid and the capsid containing the RGD in the fiber shaft poorly infected MenSCs even at high MOI, the capsid with the RGD in the HI-loop of the fiber allowed an efficient infection even at the low MOI of 5 TU/cell. A possible explanation for these results is the absence of the coxsackievirus B and adenovirus receptor (CAR) (required for the initial attachment of Ad5 to cell surface) and the presence of *α*v-integrins (required for the second interaction through the RGD motif located on the penton base protein of the capsid) on the surface of MenSCs (Figure 2(b)). Therefore, whereas MenSCs lack the primary receptor of wild-type and RGDK capsids (AdGL and AdGLK), the insertion of the RGD in the fiber knob (AdGLRGD) allows the efficient use of integrins as a primary receptor instead of CAR, increasing MenSC infectivity.

We next evaluated the ability of MenSCs to allow adenovirus replication.  Figure 2(c) shows the production of new viral particles from MenSCs, BM-MSCs, and A549 (a highly permissive epithelial tumor cell line commonly used for production of oncolytic adenoviruses) infected with ICOVIR15. The MOI used for MSC was 50 TU/cell (to ensure 100% infection) whereas for A549, the MOI was 10 TU/cell. Although the kinetics of viral production in MenSCs and BM-MSCs is delayed in the first 24 h compared to A549, the total production yield after 72 h is only 1.5-fold lower in MenSCs and 1.6-fold lower in BM-MSCs than in A549 (2237 TU per A549 cell versus 1455 TU per MenSCs and 1362TU for BM-MSCs). These results clearly demonstrate that MenSCs are permissive for oncolytic adenovirus replication.

Once the capability of infected MenSCs to generate new adenovirus particles is demonstrated, we next tested whether an oncolytic adenovirus produced within MenSCs could kill different cancer cell lines in an in vitro coculture system. MenSCs were infected with ICOVIR15 at a MOI of 50 TU/cell for 24 h. The next day, infected MenSCs were cocultured with cancer cell lines at different infected MenSCs : cancer cell ratios. As shown in Figure 2(d), coculture of infected MenSC with cancer cell lines resulted in an efficient death of cancer cell lines after 5 days of coculture, with an IC50 (number of infected MenSCs necessary to eliminate 50% of cancer cells) ranging from 0.0047 to 0.086.

### 3.4. MenSCs Infected with an Oncolytic Adenovirus Migrate to Subcutaneous Xenograft Tumor In Vivo

**Figure 3:**
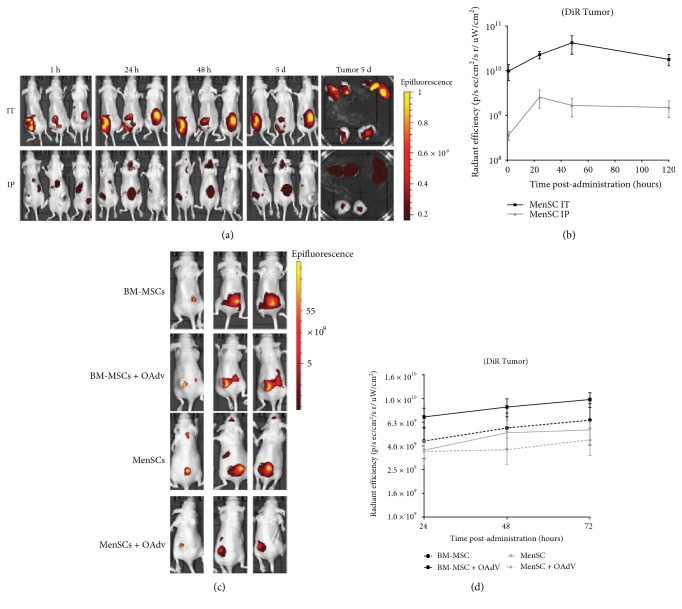
Tumor tropism of MenSCs. (a) A549 tumor-bearing immunodeficient mice (*n* = 3) were administered with 1 × 10^6^ DiR-labeled MenSCs intratumorally (IT) or intraperitoneally (IP), and fluorescence signal was detected using an in vivo imaging system (IVIS) at the indicated time points after cell administration. At the end of the experiment (5d), tumors were harvested and exposed to fluorescence analysis. (b) Intensity of fluorescence in tumors was quantified with the IVIS living image software. Mean values (*n* = 3) ± SD are represented. (c) To evaluate the impact of OAdv infection on tumor-homing capacity of MenSCs and BM-MSCs, A549 tumor-bearing immunodeficient mice (*n* = 5) were injected intraperitoneally with 1 × 10^6^ of BM-MSCs (previously labeled with DiR), MenSCs (previously labeled with DiR), BM-MSCs-OAdv (BM-MSCs previously infected with ICOVIR15 at MOI 50 for 2 h and labeled with DiR), and MenSCs-OAdv (MenSCs previously infected with ICOVIR15 at MOI 50 for 2 h and labeled with DiR). Fluorescence signal was detected using IVIS at the indicated time points after cell administration. (d) Intensity of tumor fluorescence was quantified with IVIS software. Mean values (*n* = 5) ± SD are plotted. No statistical differences in tumor fluorescence values were observed between mesenchymal from different origins (bone marrow versus menstrual blood) and between infected and noninfected cells for each cell type (BM-MSC versus BM-MSC + OAdv and MenSC versus MenSC + OAdv) at any time points analyzed (Mann–Whitney *U* test).

To test the homing capacity of MenSCs to subcutaneous tumors in vivo after regional administration (intraperitoneal), 1 × 10^6^ DiR-labeled MenSCs were administered intraperitoneally or intratumorally in A549 tumor-bearing immunodeficient mice. One hour after administration, fluorescent signal was detected in the tumors of all treated animals, showing approximately a 10-fold higher signal after 24 h without significant variations until day 5 when animals were sacrificed (Figure 3(a)). As expected, fluorescence signal in tumors administered intratumorally was higher than that administered intraperitoneally. However, the presence and enrichment of MenSCs in the tumor after intraperitoneal administration (the fluorescence signal at day 5 was 4.2 times higher than at 1 h postadministration) demonstrate the tumor tropism of MenSCs in this model (Figure 3(b)).

One concern regarding the use of MSCs as cell carriers for adenovirus delivery to tumors is a possible loss of tumor tropism due to virus infection. We compared the homing capability to subcutaneous xenograft tumors of infected and uninfected MenSCs and BM-MSCs. A549 tumor-bearing mice were injected intraperitoneally with 1 × 10^6^ of DiR-labeled BM-MSCs, MenSCs, BM-MSCs-OAdv (BM-MSCs previously infected with ICOVIR15 at MOI 50 for 2 h), and MenSCs-OAdv (MenSCs previously infected with ICOVIR15 at MOI 50 for 2 h), and cells were imaged at 24 h, 48 h, and 72 h postadministration. As shown in Figure 3(c), labeled cells from all groups could be detected in tumors at 24 h postadministration, with a higher signal at 48 h and 72 h (not significant) suggesting a progressive accumulation of the administered cells in tumors over time. Quantification of fluorescence signal at the different time points (Figure 3(d)) demonstrates the same capability of tumor homing for BM-MSCs and MenSCs, independent of oncolytic adenovirus infection. The slight difference (not statistically significant) between uninfected versus infected BM-MSCs and uninfected versus infected MenSCs (solid line versus dotted line for each cell type) was probably due to the progressive replication of the virus in the infected MSCs that led to cell lysis.

## 4. Discussion

In the present work, we have studied the phenotype and growth properties of MenSCs isolated and cultured from diverse donors. We have confirmed that isolated MenSCs are morphologically very similar to bone marrow MSC, showing the classical spindle-shaped fibroblast-like morphology of MSCs, as previously described [21]. Moreover, the immunophenotypic analysis revealed their mesenchymal stem cell nature, and their capacity to differentiate into various mesodermal cell types (adipocytes, chondrocytes, and osteocytes) confirmed their multipotency attribute which is also a characteristic of MSCs [33].

The proliferative potential of MenSCs versus BM-MSCs has been also evaluated. Through direct comparison experiments, we have determined that MenSCs had a greater growth potential than BM-MSCs, not only because of their higher clonogenic capability but also because of their shorter population DT and their near to exponential cell growth kinetics. Furthermore, we did not observe any inverse correlation between the donor age and the number of MSC isolated from the menstrual blood sample or the growth potential, contrary to what has been described for bone marrow-derived mesenchymal stem cells [34]. These features also represent an advantage in using MenSCs rather than MSCs from other sources, especially when high amount of cells are needed in a short period of time.

Infectivity, viral replication and release of new viral particles are required properties for MenSCs in order to their application as cell carriers for oncolytic adenoviruses. These features were evaluated in the present work step by step. First, we analyzed the infectivity of MenSCs by three different capsid-modified adenovirus vectors. Our results indicate that while wild-type capsid poorly infects MenSCs, virus carrying an extra RGD motif in the HI-loop of the fiber knob can efficiently infect MenSCs, even at low MOIs. As previously described for BM-MSCs [35], the analysis of adenovirus serotype 5 receptors in the surface of MenSCs showed the absence of the coxsackievirus B and adenovirus receptor (CAR), and the presence of *α*v-integrins, indicating that the insertion of the RGD in the fiber knob probably allows the use of integrins as a primary receptor instead of CAR, which could explain the increase of infectivity by this modified capsid. We next evaluated the OAdv production in MenSCs compared with BM-MSCs and A549 cells, demonstrating a similar viral production and release from MenSCs and BM-MSCs, being only 1.5 times lower than A549. Last, the oncolytic capacity of the viral progeny released from infected MenSCs was demonstrated in vitro, as culturing 1 infected MenSC for each 11(SKmel28)-213(A549) tumor cell (numerical transformation from IC_50_ established from this experiment (1 infected MenSC/IC_50_) = number of tumor cells) could eliminate 50% of cancer cells in 5 days. Altogether, these results represent, to our knowledge, the first report demonstrating the capability of MenSCs to generate and release functional oncolytic adenovirus particles.

Tumor homing for OAdv-infected MSCs has been reported not only after local administration as intracranial [36, 37] or intraperitoneal [38, 39], but also after intravenous administration in orthotopic mouse models [40] and subcutaneous xenograft mouse models [41, 42]. To study if MenSCs retain this tumor tropism observed in other MSCs, we evaluated MenSC migration in a subcutaneous xenograft tumor model after intraperitoneal administration. The intravenous administration was not considered for our experimental design since MSCs are retained in the lung due to their size [43], and considering that adenovirus replication cycle is completed after 72–96 h, the virus would kill the cells before reaching the tumors. Our in vivo results show that, in our tumor model, both MenSCs and BM-MSCs display the same tumor-homing properties, which are not affected by OAdv infection.

Altogether, we show that MenSCs represent a realistic alternative to BM-MSCs as OAdv cell carriers. Through direct comparison, we demonstrate that they share the same properties regarding viral amplification and tumor homing. In addition, the switch from bone marrow to menstrual blood as source for MSCs involves important advantages including (i) wide availability since millions of potential donors could be used periodically; (ii) low cost, as no clinical intervention or hospitalization is needed; and (iii) faster, due to their increased growth kinetics. MenSCs face, however, limitations in applications requiring autologous MSCs, since only women before menopause could be considered as donors.

Finally, in addition to the potential of MenSCs as surrogate of BM-MSCs, they can also be considered as an alternative to the use of naked OAdv. The use of cell carriers for OAdv could represent an advantage in those cases where the use of naked OAdv would be especially challenging, such as in patients with high levels of anti-Ad5 neutralizing antibodies (NAbs) or when readministration is required (usually not effective due to the immunity generated by the first administration). Theoretically, hiding the virus to the immune system using cell carriers could avoid the neutralization of the virus by NAbs, leading to a higher antitumor efficacy. Despite this protection from NAbs still needs to be demonstrated, an increase in the viral persistence has been reported after administration of OAdv-loaded bone marrow-derived mesenchymal stem cells in an immunocompetent model, associated with the capacity of MSCs to immunosuppress the antiviral immune response [44].

## 5. Conclusions

Menstrual blood-derived mesenchymal stem cells can be easily isolated, amplified, and infected with different Ad5-based capsid-modified adenoviruses. Adenovirus-infected menstrual blood-derived mesenchymal stem cells produce and release viral progeny and home tumors upon regional administration.

The use of menstrual blood-derived mesenchymal stem cells as carriers for oncolytic adenoviruses to human tumors warrants further testing towards clinical application.

## Supplementary Material

Supplementary Figure 1. Morphologic and phenotypic characterization of Menstrual Blood-Derived Mesenchymal Stem Cells. A) Phase-contrast microscopy image of mesenchymal stem cells isolated from menstrual blood (MenSCs). Cells show a spindle-shaped fibroblast-like morphology. The MenSCs population was induced with the respective differentiation media for 3–4 weeks. B) The lipid droplets present into adipocytes were stained with Oil Red-O (left, control non-differentiated stained MenSCs, right). C) Chondrocyte mucopolysaccharides were observed with Alcian Blue staining (left, control non-differentiated stained MenSCs, right). The calcification of osteocytes was observed through Alizarin Red staining (left, control non-differentiated stained MenSCs, right) D). Magnification 20x; scale bar: 10μm. Supplementary Figure 2. Representative flow cytometry analysis of MenSCs immunophenotype. While MenSCs strongly express mesenchymal stem cell antigens (CD29, CD90, CD44, CD73 and CD105) they do not express neither hematopioetic markers (CD34, HLA-DR, CD133 and CD14) nor co-stimulatory proteins (CD40, CD83, CD86).
